# Supplementary material for: Registration and reporting of clinical trials affiliated with California universities and with primary completion date from 2014 to 2017
Source: Trials. 2025 Dec 1;27:4. doi: 10.1186/s13063-025-09270-2 (PMC12771935; doi:10.1186/s13063-025-09270-2)
Supplement: Supplementary file 1 — Supplementary Material 1. Appendix. [file 13063_2025_9270_MOESM1_ESM.docx]

Appendix for ***Registration and Reporting of Clinical Trials Affiliated with California Universities and with Primary Completion Date from 2014 to 2017*** by Mario Malički, Vladislav Nachev, Susanne Wieschowski, Nicole Hildebrand, Stefanie Gestrich, Samruddhi Yerunkar, Emmanuel Zavalis, Benjamin Gregory Carlisle, Delwen L. Franzen, Maia Salholz-Hillel, Steven N. Goodman, Daniel Strech.

Results are presented in the order they appear in the manuscript. All data was explored using MedCalc v.20.123 (RRID:SCR_015044) or Microsoft Excel v.2211 (RRID:SCR_016137).

Table of Contents

[Inter-rater agreement 2](#_Toc211964520)

[Extraction of Dates 2](#_Toc211964521)

[Results 3](#_Toc211964522)

[Appendix Table 1. 3](#_Toc211964523)

[Study Dates 3](#_Toc211964524)

[Trial Registration 3](#_Toc211964525)

[Publication Searches 4](#_Toc211964526)

[Appendix Table 2. 4](#_Toc211964527)

[Appendix Table 3. 5](#_Toc211964528)

[Appendix Table 4. 5](#_Toc211964529)

[Appendix Table 5. 6](#_Toc211964530)

# Inter-rater agreement

While initially 3 raters had slightly lower results than the prespecified threshold (2 77%, and one 67%), we included all raters in the extraction process, as raters’ mistakes were related to trials having more than one publication with results, and raters not choosing the earliest one - rather than stating there was no publication when there was one. Therefore, the final results might slightly impact the time to publication rather than the rates of publications.

# Extraction of Dates

Trial start date, registration date and primary completion year date were extracted from the Clinical Trials Transformation Initiative’s Aggregate Content of ClinicalTrials.gov (AACT) database. The majority of trials did not contain information on the day of the month.

| **Variable** | **n (%)** |
| --- | --- |
| Study start date | 913 (84) |
| Study primary completion date | 765 (70) |
| Publication date | 7 (1) |

Publication date information came from 3 sources: manual extraction, PubMed, or Unpaywall. Dates from PubMed were retrieved in the following way: we queried PubMed with the list of all PMIDs (separated with a blank space) and then exported the results as a csv file. From the citation variable of the csv export, we then extracted the publication dates and the e-publication dates (if available).

If a paper had only a manually extracted publication date, that date was used for all date comparisons. If a publication also had dates retrieved from Unpaywall and PubMed, we took the earliest date associated with it as the publication date (either electronic or print publication date from PubMed or the date given on Unpaywall). Publication dates listed on Unpaywall without the exact day of the month were disregarded.

# Results

Appendix Table 1. **Trial phases.**

| **Phase** | **n** | **%** |
| --- | --- | --- |
| Early Phase 1 | 25 | 2% |
| Not Applicable | 589 | 54% |
| Phase 1 | 103 | 9% |
| Phase 1/Phase 2 | 54 | 5% |
| Phase 2 | 159 | 15% |
| Phase 2/Phase 3 | 18 | 2% |
| Phase 3 | 64 | 6% |
| Phase 4 | 79 | 7% |
| **Total** | **1091** | **100.0%** |

## Study Dates

ClinicalTrials.gov allowed entering only year and month for dates related to study start, registration, and completion. We obtained these three dates from the AACT database. 84% of studies had month/year dates for study start, 70% for primary completion date, and 68% for completion date. For all three of these dates, the likelihood of having complete vs. month/year dates differed significantly between the universities, by primary completion year, and by study start year (data not shown).

## Trial Registration

The results for prospective registration presented in the manuscript results were calculated using the month method (see details in manuscript methods). We also evaluated registration allowing for 21 days following the study start date (see methods) and limited to trials with exact dates (i.e., not month/year only) (see Study Dates, above). Overall, 59% of trials (n=641) were registered prospectively or within 21 days of study start. Prospective registration statistically differed between universities (P<0.0001, chi-square test, Table 1), and per-trial primary completion year, growing from 49% for trials with primary completion in 2014 to 69% in those with primary completion date in 2017 (P=0.0097, chi-square for trend).

Limiting to trials with exact dates (i.e., not month/year only) for study start (N=178), 56% were registered on or before the study start date, which increased to 63% when allowing for registration within 21 days of study start.

## Publication Searches

We found three duplicate publications (2 from Stanford and 1 from UCSF, of which 1 was hybrid, 1 closed, and 1 bronze) that listed 2 trial registrations each. Thus, the total number of unique publications was 747. Of these, 2 publications were not on Unpaywall but were manually assessed for OA.

Appendix Table 2. **Summary Results reported in ClinicalTrials.gov registry for trials with primary completion date from 2014 to 2017 affiliated with seven California universities.**

| **PI and collaborator affiliation (n, %)**  **(Affiliation does not reflect legal responsibility)*** | **Summary results in ClinicalTrials.gov** | **Summary results in ClinicalTrials.gov within 2 years** | **Summary results in ClinicalTrials.gov within 5 years** |
| --- | --- | --- | --- |
| Stanford University (N=266) | 160 (60) | 104 (39) | 157 (59) |
| UC Davis (N=110) | 37 (34) | 24 (22) | 37 (34) |
| UC Irvine (N=41) | 25 (61) | 17 (41) | 23 (56) |
| UC Los Angeles (N=188) | 74 (39) | 57 (30) | 73 (39) |
| UC San Diego (N=127) | 68 (54) | 31 (24) | 61 (48) |
| UC San Francisco (N=299) | 117 (39) | 65 (22) | 106 (35) |
| University of Southern California (N=81) | 28 (35) | 21 (26) | 27 (33) |
| Total (N=1112)** | 509 (46) | 319 (29) | 484 (44) |
| P (chi-squared test) | < 0.0001 | 0.0001 | < 0.0001 |

*We defined a trial as affiliated with a university if the university or one of its investigators was mentioned in one of the following fields of a clinicaltrials.gov registry entries "Overall Official," "Responsible Parties," or "Sponsors." See Methods for more details.

**Trials involving individuals from more than one university were counted for each university (i.e., there were 1091 unique trials, of which 1072 were classified as involving 1 California university, 17 involving two California universities, and 2 involving 3 California universities).

Appendix Table 3. **Publication rates for trials with primary completion date from 2014 to 2017 affiliated with seven California universities.**

| **PI and collaborator affiliation (n, %)**  **(Affiliation does not reflect legal responsibility)*** | **Journal publication** | **Abstract/Poster** | **Publication within 2 years** | **Publication within 5 years** | **Open Access*** |
| --- | --- | --- | --- | --- | --- |
| Stanford University (N=266) | 173 (65) | 4 (2) | 116 (44) | 172 (65) | 122 (69) |
| UC Davis (N=110) | 82 (75) | 4 (4) | 57 (52) | 81 (74) | 63 (73) |
| UC Irvine (N=41) | 23 (56) | 3 (7) | 15 (37) | 25 (61) | 18 (69) |
| UC Los Angeles (N=188) | 128 (68) | 2 (1) | 83 (44) | 125 (66) | 108 (83) |
| UC San Diego (N=127) | 83 (65) | 5 (4) | 56 (44) | 85 (67) | 71 (81) |
| UC San Francisco (N=299) | 228 (76) | 13 (4) | 152 (51) | 231 (77) | 188 (78) |
| University of Southern California (N=81) | 52 (64) | 5 (6) | 38 (48) | 53 (65) | 35 (61) |
| Total (N=1112)** | 769 (69) | 36 (3) | 518 (47) | 772 (69) | 605 (75) |
| P (chi-squared test) | 0.0026 | | 0.3377 | 0.0165 | 0.0094 |

*We defined a trial as affiliated with a university if the university or one of its investigators was mentioned in one of the following fields of a clinicaltrials.gov registry entries "Overall Official," "Responsible Parties," or "Sponsors." See Methods for more details.

**Trials involving individuals from more than one university were counted for each university (i.e., there were 1091 unique trials, of which 1072 were classified as involving 1 California university, 17 involving two California universities, and 2 involving 3 California universities).

Appendix Table 4. **Any Results Dissemination for trials with primary completion date from 2014 to 2017 affiliated with seven California universities.**

| **PI and collaborator affiliation (n, %)**  **(Affiliation does not reflect legal responsibility)*** | **Any Result Dissemination** | **Any Result Dissemination within 2 years** | **Any Result Dissemination within 5 years** |
| --- | --- | --- | --- |
| Stanford University (N=266) | 227 (85) | 159 (60) | 224 (84) |
| UC Davis (N=110) | 92 (84) | 62 (56) | 87 (79) |
| UC Irvine (N=41) | 36 (88) | 24 (59) | 34 (83) |
| UC Los Angeles (N=188) | 147 (78) | 105 (56) | 141 (75) |
| UC San Diego (N=127) | 109 (86) | 73 (57) | 104 (82) |
| UC San Francisco (N=299) | 263 (88) | 178 (60) | 255 (85) |
| University of Southern California (N=81) | 65 (80) | 50 (62) | 62 (77) |
| Total (N=1112)** | 939 (84) | 651 (59) | 907 (82) |
| P (chi-squared test) | 0.1146 | 0.9620 | 0.0811 |

*We defined a trial as affiliated with a university if the university or one of its investigators was mentioned in one of the following fields of a clinicaltrials.gov registry entries "Overall Official," "Responsible Parties," or "Sponsors." See Methods for more details.

**Trials involving individuals from more than one university were counted for each university (i.e., there were 1091 unique trials, of which 1072 were classified as involving 1 California university, 17 involving two California universities, and 2 involving 3 California universities).

Appendix Table 5. **Publication links and trial registration number reporting for trials with primary completion date from 2014 to 2017 affiliated with seven California universities.**

| **PI and collaborator affiliation (n, %)**  **(Affiliation does not reflect legal responsibility)*** | **Journal publication** | **Publications linked in ClinicalTrials.gov** | **Trial Registration Number in Abstract** | **Trial Registration Number in Publication** |
| --- | --- | --- | --- | --- |
| Stanford University (N=266) | 173 (65) | 98 (57) | 44 (25) | 73 (43) |
| UC Davis (N=110) | 82 (75) | 48 (59) | 37 (45) | 41 (50) |
| UC Irvine (N=41) | 23 (56) | 11 (48) | 5 (22) | 6 (26) |
| UC Los Angeles (N=188) | 128 (68) | 81 (63) | 49 (40) | 58 (48) |
| UC San Diego (N=127) | 83 (65) | 50 (60) | 37 (46) | 29 (36) |
| UC San Francisco (N=299) | 228 (76) | 137 (60) | 89 (40) | 107 (49) |
| University of Southern California (N=81) | 52 (64) | 25 (48) | 15 (31) | 23 (47) |
| Total (N=1112)** | 769 (69) | 450 (59) | 276 (37) | 337 (45) |
| P (chi-squared test) | 0.0026 | 0.5188 | 0.0031 | 0.1714 |

*We defined a trial as affiliated with a university if the university or one of its investigators was mentioned in one of the following fields of a clinicaltrials.gov registry entries "Overall Official," "Responsible Parties," or "Sponsors." See Methods for more details.

**Trials involving individuals from more than one university were counted for each university (i.e., there were 1091 unique trials, of which 1072 were classified as involving 1 California university, 17 involving two California universities, and 2 involving 3 California universities).
